# Persistent weekly paclitaxel-induced peripheral neuropathy in early breast cancer patients enrolled in a randomized trial of cryotherapy

**DOI:** 10.1097/MD.0000000000033580

**Published:** 2023-04-21

**Authors:** Hideo Shigematsu, Yuri Kimura, Tomoko Itagaki, Daisuke Yasui

**Affiliations:** a Department of Breast Surgery, National Hospital Organization Kure Medical Center and Chugoku Cancer Center, 3-1, Aoyama-cho, Kure-City, Hiroshima 737-0023, Japan.

**Keywords:** breast cancer, cryotherapy, paclitaxel, persistent CIPN

## Abstract

Chemotherapy-induced peripheral neuropathy (CIPN) is a serious side effect of weekly paclitaxel-based chemotherapy for breast cancer, that can persist for years. Cryotherapy therapy is effective for preventing early CIPN, but its protective effect on persistent CIPN is uncertain. This is a cross-sectional study conducted as an ancillary analysis of a randomized trial investigating the preventive effect of cryotherapy on CIPN in breast cancer patients receiving weekly paclitaxel-based chemotherapy (UMIN000034966). Eligible patients were evaluated for CIPN at more than a year after completion of the chemotherapy (persistent CIPN). CIPN was defined as a 6 or more points reduction from baseline in the Functional Assessment of Cancer Therapy-Neurotoxicity (FACT-NTX) score. The incidence of early and persistent CIPN was compared between cryotherapy and control groups. Thirty-eight patients were examined for both early and persistent CIPN. The median time from completion of the weekly paclitaxel-based chemotherapy to the questionnaire for persistent CIPN was 2.3 (1.3–3.1) years. In all 38 patients, persistent CIPN was demonstrated in 10 (26.3%), respectively. There was a numerical, however not significant, reduction in the incidence of persistent CIPN (15.8% vs 36.8%, *P* = .1) in the cryotherapy group compared with the control group, respectively. In multivariate logistic regression analysis, age ≥ 65 was a substantial risk factor for persistent CIPN (HR: 14.7, 95%CI: 1.7–130.7, *P* = .01). In breast cancer patients receiving adjuvant weekly paclitaxel-based chemotherapy, cryotherapy resulted in a numerical, however not significant, reduction in the incidence of persistent CIPN and age>=65 was a risk factor for persistent CIPN.

## 1. Introduction

Chemotherapy-induced peripheral neuropathy (CIPN) is a serious adverse event that significantly deteriorates the quality of life and survivorship.^[1]^ Weekly paclitaxel-based chemotherapy is one of the standard treatments for early breast cancer, but this regimen is linked to the high incidence and severity of CIPN.^[2]^ Although paclitaxel related CIPN improves over time, a significant proportion of breast cancer patients experienced persistent CIPN in breast cancer patients. A cross-sectional study of 50 consecutive early breast cancer patients found that 81% of the patients had symptoms of CIPN and severe symptoms were reported in 27% of patients at a median of 12 months after the end of paclitaxel-based chemotherapy.^[3]^ A prospective cohort study of 50 early breast cancer patients also found that 67% had persistent numbness in their hands or feet, including 27% with severe symptoms at 12 months after finishing paclitaxel-based chemotherapy.^[3]^ Another cohort study also found that CIPN persisted in 64 and 41% of early breast cancer patients at 1 and 3 years after starting adjuvant paclitaxel-based chemotherapy, respectively.^[4]^ Given the recent improvement in breast cancer prognosis, it is critical to address the prevention and treatment of paclitaxel-induced persistent CIPN.

For patients with paclitaxel-induced persistent CIPN, there is weak evidence for treatment to relieve the symptoms. Duloxetine is the only recommended treatment for symptomatic CIPN, however, duloxetine may be less effective for taxane-induced than oxaliplatin-induced CIPN.^[5]^ Other trials failed to demonstrate the efficacy of duloxetine over pregabalin in patients with taxane-induced CIPN.^[6]^ Aside from drug therapy, physical therapies including scrambler therapy, acupuncture, and exercise may be effective in patients with persistent CIPN considering their various benefits and limited harms,^[7–9]^ but guidelines concluded that no recommendation about the use of these physical therapies for patients with persistent CIPN could be made.^[1,10]^

Compared with the treatment of persistent CIPN, physical therapy, prevention of CIPN with cryotherapy, compression therapy and exercise may be more effective modalities in patients receiving neurotoxic chemotherapy. Cryotherapy and compression therapy are thought to prevent acute CIPN by reducing paclitaxel exposure in fingertip and toes through their analogous mechanism of compression or vasoconstriction.^[11,12]^ In a prospective self-controlled trial evaluating the effect of frozen gloves and socks on weekly paclitaxel related-CIPN, a reduction in tactile sensation was observed in 28 and 25% of patients in treated hands and feet, respectively.^[11]^ A phase II multicenter study of compression therapy found that treated hands had a lower incidence of CTCAE grade2 or higher of sensory (21.4% vs 76.1%) and motor (26.2% vs 57.1%) peripheral neuropathies than control hands.^[12]^ Exercise is also advised for patients receiving neurotoxic chemotherapy considering its various benefits, including its CIPN preventive and mitigative effects.^[13,14]^ Although these physical therapies may reduce the incidence of CIPN during chemotherapy, their preventive effects on persistent CIPN years later have not been studied.

The study aims to assess the preventive effect of cryotherapy on persistent CIPN at more than 1 year after completing weekly paclitaxel-based chemotherapy. We conducted a cross-sectional study as an ancillary analysis of the previous trial that assessed the preventive effect of cryotherapy on paclitaxel-induced early CIPN in breast cancer patients (UMIN000034966).^[15]^ In this research, we compared the incidence of persistent CIPN between cryotherapy and control group in breast cancer patients receiving weekly paclitaxel-based chemotherapy.

## 2. Materials and Methods

### 2.1. Study design and participants

This cross-sectional study was conducted performed as adjunct to a randomized trial evaluating the preventive effect of cryotherapy on weekly paclitaxel-induced CIPN in breast cancer patients (UMIN000034966). The original randomized trial detailed information and results are described in a previous report.^[15]^ Briefly, the main inclusion criteria were a diagnosis of invasive breast cancer scheduled for a regimen of 12 weekly doses of paclitaxel and the main exclusion criteria were presence of grade ≥ 2 CIPN or current systemic treatment for CIPN. Most patients received weekly paclitaxel-based chemotherapy as adjuvant systemic chemotherapy for stage I to III stage breast cancer. In the cryotherapy group, patients wore −20°C frozen gloves/socks (Elasto-Gel) on both hands and feet continuously during the paclitaxel infusion throughout the 12 weekly paclitaxel. Regarding this study, the inclusion criteria of this study are the following; enrolled in the prior clinical trial (UMIN000034966) and able to complete the CIPN questionnaire at more than a year after completing paclitaxel-based chemotherapy. Exclusion criteria are the following: having neurotoxic chemotherapy for advanced or recurrent breast cancer. Disqualified to respond to a questionnaire for CIPN and withdrawal of consent from the previous study. There was no special requirement for treatment to alleviate CIPN-related symptoms after completion of cryotherapy. Patients were divided into cryotherapy and control groups according to the administration of cryotherapy during adjuvant weekly paclitaxel-based chemotherapy. Cryotherapy and control group incidences of early and persistent CIPN were compared. Risk factors for both early and persistent CIPN were also investigated. This observational study was approved by the Ethics Committee of the National Hospital Organization of Kure Medical Center and Chugoku Cancer Center (Approval number 28–70) and followed the Helsinki Declaration and the ethical principles for clinical research. All patients received a thorough explanation of the study from their primary care physician and informed consent was obtained before enrollment.

### 2.2. Evaluation of CIPN

Eligible patients were assessed for early and persistent CIPN using a questionnaire survey for Functional Assessment of Cancer Therapy-Neurotoxicity (FACT-NTX) score at treatment completion (evaluated as early CIPN) and more than 1 year after treatment completion (evaluated as persistent CIPN). Briefly, FACT-NTX has 11 items assessing CIPN-related symptoms with a total score range from 0 to 44. A lower FACT-NTX score indicates worse peripheral neuropathy.^[16]^ A 6-point or more decrease in the total FACT-NTX score from baseline is defined as clinically significant CIPN.^[17]^ The questionnaire for CIPN was performed between April 2020 and October 2020. The median time from completion of the weekly paclitaxel-based chemotherapy to the questionnaire for persistent CIPN in this research was 2.3 (1.3–3.1) years.

### 2.3. Statistical analysis

Groups assessed patient characteristics using a Fisher exact test for categorical variables and a *t* test for continuous variables. A Fisher exact test was used to contrast the incidence of early and persistent CIPN between the cryotherapy and control groups. A 2-sided *P* value < .05 was considered substantial. A multivariate logistic regression analysis was used to assess predictive factors for early and persistent CIPN. Statistical analyses were conducted using JMP software version 13.2.1 (SAS Institute Inc., Carys).

## 3. Results

### 3.1. Patient characteristics

**Figure 1.**
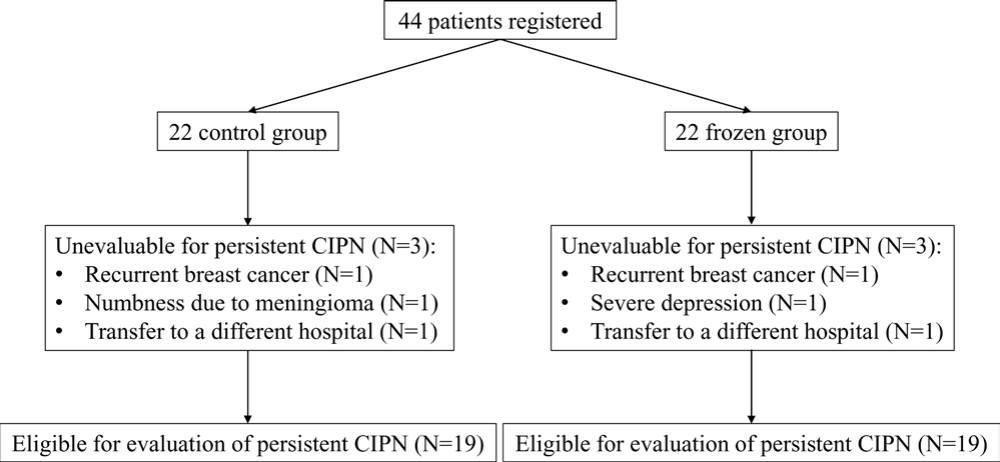
Consort diagram for this survey.

Of the 44 patients enrolled in the previous randomized trial, 38 patients met the eligibility criteria. Six patients were exempted from this survey due to the following reasons: having neurotoxic therapy for advanced or recurrent breast cancer,^[18]^ transfer to a different hospital,^[18]^ severe depression,^[16]^ and presence of severe neuropathy due to surgery for meningioma^[16]^ (Fig. 1). In this study, 19 patients were cryotherapy group and 19 were the control group. Among 38 patients, 34 patients completed 12 weekly paclitaxel-based chemotherapy with cumulative dose of paclitaxel 960mg/m^2^, and 3 patients in the control group and 1 patient in the cryotherapy group could not complete 12 weekly paclitaxel therapy due to adverse events. In 19 cryotherapy group, 6 patients showed incomplete compliance to cryotherapy. There was no statistical variation in baseline clinicopathological factors, including age, height, weight, FACT-NTX score at baseline, stage, subtype, adjuvant endocrine therapy, radiation therapy and interval from completion of paclitaxel-based chemotherapy between the cryotherapy and control group (Table 1).

**Table 1:** Clinicopathological factors.

Factors		Cryotherapy(n = 19)	Control(n = 19)	*P* value*
Age, yr	65>	5	14	.7
	65=<	4	15	
Height, mean (SD), cm		156.5 (5.9)	154.1 (7.1)	.3
Weight, mean (SD), kg		52.3 (7.1)	55.9 (9.1)	.2
FACT-NTX score at baseline, mean (range)		1 (0–10)	1 (1–6)	1
Stage	I	4	2	.7
	II	8	9	
	III	7	8	
Subtype	luminal	3	5	1
	HER2	12	11	
	TNBC	4	3	
Completion of adjuvant 12 weekly paclitaxel	yes	18	16	.6
	no	1	3	
Adjuvant endocrine therapy	yes	15	16	1
	no	4	3	
Adjuvant radiation therapy	yes	14	15	1
	no	5	4	

FACT-NTx = functional assessment of cancer therapy-neurotoxicity, SD = standard deviation, TNBC = triple negative breast cancer.

* Factors were compared by treatment groups using Fisher exact test for categorical variables and *t* tests for continuous variables. Tests were 2-sided.

### 3.2. Effect of cryotherapy on the incidence of persistent CIPN

**Figure 2.**
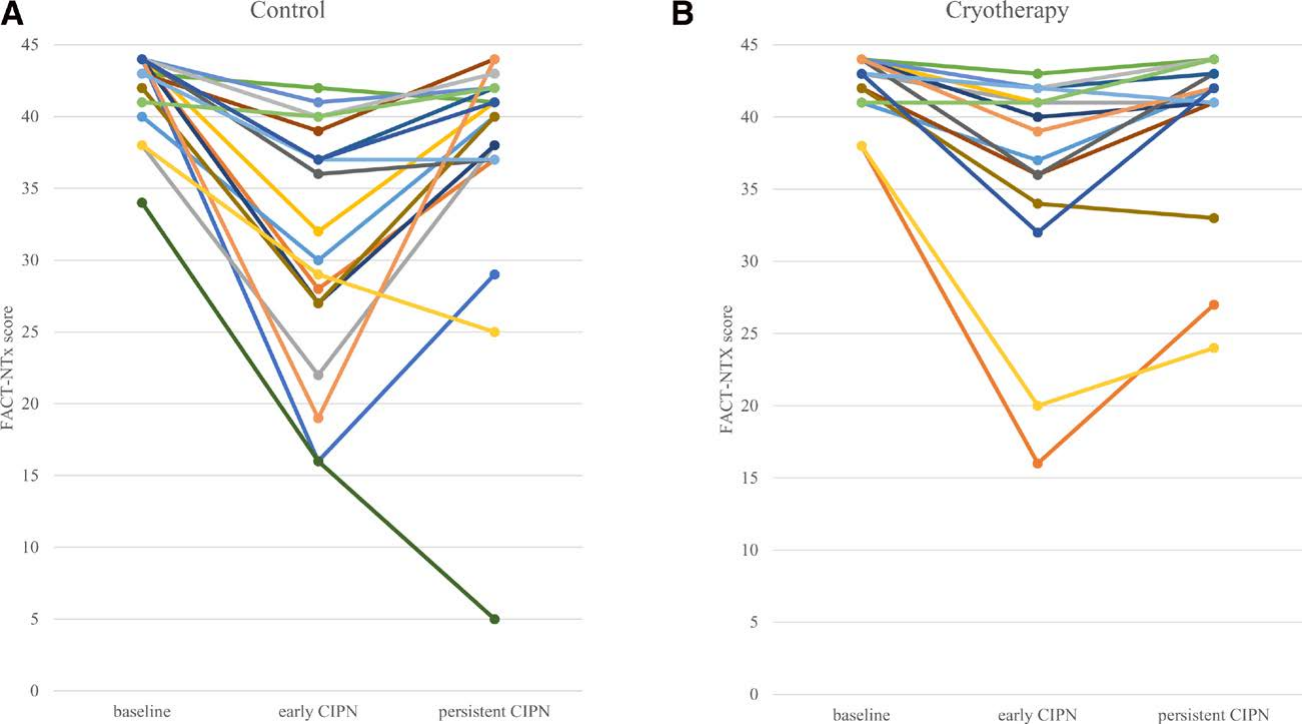
The individual FACT-NTX scores at baseline, completion of weekly paclitaxel-based chemotherapy (early CIPN), and more than 1 year after the treatment completion (persistent CIPN) in the (A) control group and (B) cryotherapy groups. CIPN = chemotherapy-induced peripheral neuropathy, FACT-NTX = functional assessment of cancer therapy-neurotoxicity.

In all 38 patients, persistent CIPN was demonstrated in 10 (26.3%). The incidence of persistent CIPN was 15.8% and 36.8% in the cryotherapy and control group, respectively, indicating a numerically but not significantly lower incidence of persistent CIPN in the cryotherapy group compared with the control group (*P* = .1). Figure 2A and B depicts the individual FACT-NTX score according to treatment arm at baseline, completion of weekly paclitaxel-based chemotherapy (early CIPN), and more than 1 year after treatment completion (persistent CIPN). In control group, median score of FACT-NTX at baseline, early CIPN and persistent CIPN was 43, 35, and 40, respectively (Fig. 2A). In cryotherapy group, median score of FACT-NTX at baseline, early CIPN, and persistent CIPN was 43, 41, and 42, respectively (Fig. 2B). Increments in FACT-NTX score from early CIPN to persistent CIPN were recognized in 15 (78.9%) and 16 (84.2%) patients in the control and cryotherapy group, respectively (Fig. 2A and B). A subset of 5 patients with baseline FACT-NTX score ≥ 6 demonstrated high FACT-NTX scores at both the early CIPN and persistent CIPN evaluations. A multivariate logistic regression analysis was used to assess the relationship between clinicopathological factors, including age, cryotherapy, height, weight, completion of adjuvant 12 weekly paclitaxel, adjuvant endocrine therapy, adjuvant radiation therapy and interval length, and the incidence of persistent CIPN (Table 2). In a multivariate logistic analysis, age ≥ 65 was a significant risk factor for persistent CIPN (HR: 18.5, 95%CI: 1.8–187.3, *P* = .01); however, no cryotherapy was not a significant factor for persistent CIPN (HR: 4.2, 95%CI: 0.6–29.5, *P* = .1).

**Table 2:** A multivariate logistic regression analysis to evaluate the association between clinicopathological factors and the incidence of persistent CIPN*.

Factors		HR	95% CI	*P* value
Age	65>	1		.01
	65=<	18.5	1.8–187.3	
Height (cm)		0.9	0.8–1.1	.4
Weight (kg)		1	0.9–1.2	.8
Completion of adjuvant 12 weekly paclitaxel	yes	1		.6
	no	2.9	0.08–106.8	
Adjuvant hormone therapy	no	1		.3
	yes	5.1	0.30–100.5	
Adjuvant radiotherapy	no	1		.4
	yes	2.5	0.3–21.7	
Interval from completion of paclitaxel-based chemotherapy (yr)		0.9	0.2–4.4	.9
Cryotherapy	yes	1		.1
	no	4.2	0.6–29.5	

CI = confidence interval, CIPN = chemotherapy induced peripheral neuropathy, HR = hazard ratio. A multivariate logistic regression analysis was used to evaluate predictive factor for acute and persistent CIPN.

* A 6-point or more decrease in the total of FACT-NTX score from baseline is regarded as occurrence of CIPN.

The other clinicopathological factors were not significant predictors of persistent CIPN. Regarding compliance to cryotherapy, persistent CIPN was recognized in 3 (50%) of 6 patients with incomplete compliance and 3 (23%) of 13 patients with complete compliance, respectively.

## 4. Discussion

In this ancillary analysis of a previous randomized trial investigating the preventive effect of cryotherapy on weekly paclitaxel-induced CIPN in breast cancer patients, cryotherapy resulted in substantially lower incidence of early CIPN and a numerical decrease in the incidence of persistent CIPN at more than 1 year after treatment completion when compared to control. Age ≥ 65 year was a substantial predictive factor for persistent CIPN in multivariate logistic regression analysis.

In early breast cancer patients administered with adjuvant taxane-based chemotherapy, CIPN is a common and prolonged adverse event that can complicate quality of life and increase the risk of falls or functional impairment. Taxane-induced CIPN is commonly recognized during chemotherapy, however, recent studies have reported the presence of persistent CIPN after treatment completion. In a randomized trial of acetyl-L-carnitine for the prevention of taxane-induced CIPN (SWOG S0715), FACT-NTX scores on the placebo and experimental arm stayed decreased during weeks 12, 24, 36, 52, and 104.^[19,20]^ The proportion of significant decreases in FACT-NTX score was not decreased during the study period. As a result, 39.5% and 34.4% of the experimental and placebo groups, respectively, reported a significant decrease in the FACT-NTX score from baseline at 104 weeks. A prospective cohort study of 50 breast cancer patients who received taxane-based adjuvant chemotherapy yielded comparable results.^[3]^ The mean scores on the FACT-NTX reduced from 37.5 to 28.7, 30.6, 31.2, 31.8, and 33.0 at post-treatment, 3, 6, 9, and 12 months after treatment, respectively. In our study of 38 patients, the FACT-NTX score improved over time, but a significant decrease in FACT-NTX score remained in 10 patients (26.3%) at a median 2.3 (1.3–3.1) years after completion of weekly paclitaxel-based chemotherapy. Furthermore, more than 1-third (36.8%) of patients in control group showed persistent CIPN. Our study is a cross-sectional analysis with various durations since the completion of paclitaxel-based chemotherapy; however, the duration was not a significant factor for persistent CIPN in multivariate logistic regression analysis. These results indicate that taxane-related CIPN can persist for years and persistent CIPN is a critical clinical issue to be resolved in breast cancer patients.

Risk factors of CIPN occurrence include chemotherapy regimen, age, comorbidities, and preexisting neuropathy. Taxane-containing regimen is well established neurotoxic chemotherapy and paclitaxel is more neurotoxic than docetaxel. Regarding patient factors, age and history of diabetes are risk factors for CIPN.^[21]^ The presence of a preexisting neuropathic disease is also a risk factor for severe CIPN with various neurotoxic chemotherapy.^[18]^ Few studies assessed risk factors of persistent CIPN, however, age appears to be a critical risk factor. A retrospective cohort study of 219 breast cancer patients treated with paclitaxel-based chemotherapy found that age 60 years or older was significantly correlated with CIPN duration compared to age < 60 (HR: 0.55, *P* value: .027).^[4]^ The SWOG S0715 trial also found age 60 years or older was a risk factor for CIPN persistence in year 1 (HR: 1.74, *P* = .02) and year 2 (HR: 1.67, *P* = .04).^[20]^ In our study, we stratified the cohort by age of 65 based on the criteria that World Health Organization characterizes the elderly persons as over 65 years old. The incidence of persistent CIPN was 17.2% and 55.6% in the age < 65 and age ≥ 65 group, respectively (*P* = .03, *t* test). In multivariate logistic regression analysis, age ≥ 65 was still significantly linked with a greater risk for persistent CIPN. Our study also found that patients with significant CIPN at baseline had a high FACT-NTX score after paclitaxel-based chemotherapy and more than 1 year after treatment completion. Existing neuropathy may be a risk factor for both occurrence and persistent CIPN. Previous research and our finding suggest that aged or preexisting neuropathic status are risk factors for persistent CIPN.

Previous clinical trials assessed the preventive effects of cryotherapy, compression therapy and exercise on CIPN during chemotherapy, but few reported the prevention effect of persistent CIPN with long-term follow-up. In our study, cryotherapy resulted in not only statistically lower incidence of early CIPN but also a numerically lower incidence of persistent CIPN when compared with the control group. It is possible that preventing CIPN during chemotherapy is linked to a lower incidence of persistent CIPN. A retrospective study found that having severe CIPN was a significant risk factor for CIPN duration.^[4]^ These finding suggest that more attention should be paid to CIPN during neurotoxic chemotherapy to reduce the incidence of persistent CIPN. More research is needed to confirm the efficacy of the preventive approach on persistent CIPN.

### 4.1. Study limitations

First, the small sample size and unplanned nature of the ancillary analysis without statistical power limit the strength and clinical impact of our findings. Second, the study assessment of persistent CIPN was based on a subjective patient questionnaire. Objective evaluation, such as the Semmes-Weinstein monofilament test, should be integrated to the robustness of our finding. Finally, while the degree of persistent CIPN appears to be fixed over time, additional serial assessments of CIPN on individual cases may strengthen our study findings.

## 5. Conclusion

In conclusion, we demonstrated that cryotherapy reduced the incidence of persistent CIPN numerically and ≥ age 66 was a risk factor for persistent CIPN in breast cancer patients receiving weekly paclitaxel-based chemotherapy. A prospective randomized trial with a long-term period is needed to confirm the preventive effect of cryotherapy on persistent CIPN.

## Author contributions

**Conceptualization:** Hideo Shigematsu, Daisuke Yasui, Yuri Kimura, Tomoko Itagaki.

**Data curation:** Hideo Shigematsu, Daisuke Yasui, Yuri Kimura, Tomoko Itagaki.

**Formal analysis:** Hideo Shigematsu, Yuri Kimura.

**Investigation:** Hideo Shigematsu, Daisuke Yasui, Yuri Kimura, Tomoko Itagaki.

**Methodology:** Hideo Shigematsu.

**Project administration:** Hideo Shigematsu.

**Validation:** Hideo Shigematsu.

**Writing – original draft:** Hideo Shigematsu.

**Writing – review & editing:** Hideo Shigematsu, Daisuke Yasui.

Proof of statistical analysis: We thank Research Associate (Lecturer) Tomoyuki Akita of the Department of Epidemiology, Infectious Disease Control and Prevention, Graduate School of Biomedical and Health Sciences, Hiroshima University (Hiroshima, Japan) for critically reviewing the statistical analysis of this study.

Language editing: This manuscript has been edited for English language, grammar, punctuation, and spelling by Enago, the editing brand of Crimson Interactive Pvt. Ltd under Advance Editing B2C.
